# Ultrasonic Structural Health Monitoring Using Fiber Bragg Grating

**DOI:** 10.3390/s18103395

**Published:** 2018-10-11

**Authors:** Qi Wu, Yoji Okabe, Fengming Yu

**Affiliations:** 1 Institute of Industrial Science, the University of Tokyo, 4-6-1 Komaba, Meguro-ku, Tokyo 153-8505, Japan; okabey@iis.u-tokyo.ac.jp (Y.O.); houmei@iis.u-tokyo.ac.jp (F.Y.); 2 State Key Laboratory of Mechanics and Control of Mechanical Structures, Nanjing University of Aeronautics and Astronautics, Yudao Street 29, Nanjing 210016, China

**Keywords:** fiber Bragg grating, ultrasonic sensor, demodulation technique, Lamb wave, structural health monitoring

## Abstract

The fiber Bragg grating (FBG) sensor, which was developed over recent decades, has been widely used to measure manifold static measurands in a variety of industrial sectors. Multiple experiments have demonstrated its ability in ultrasonic detection and its potential in ultrasonic structural health monitoring. Unlike static measurements, ultrasonic detection requires a higher sensitivity and broader bandwidth to ensure the fidelity of the ultrasonic Lamb wave that propagates in a plate-like structure for the subsequent waveform analysis. Thus, the FBG sensor head and its corresponding demodulation system need to be carefully designed, and other practical issues, such as the installation methods and data process methods, should also be properly addressed. In this review, the mature techniques of FBG-based ultrasonic sensors and their practical applications in ultrasonic structural health monitoring are discussed. In addition, state-of-the-art techniques are introduced to fully present the current developments.

## 1. Introduction

The health status of a structure such as an aerospace structure is always of concern to users because an initially small defect in a material may grow into large damage and lead to the catastrophic failure of the whole structure, which may even threaten people’s lives. For example, a flaw revealed by ultrasonic tests in the composite tail was thought to be a possible reason for the ply debonding and consequent tail fin separation, which led to the Airbus A300 aircraft accident in November 2001 that killed 265 people [1]. Another example is the Airbus A310 accident in March 2005, which was possibly caused by the pre-existing debonding on the rudder [2]. In the following investigation, the Transportation Safety Board found that the inspection program of composite rudders was inadequate. Thus, structural health monitoring (SHM) is eagerly required by the industry to alert the initial damage, monitor its subsequent progress, and predict the remaining life of a structure. Compared to conventional static SHM, ultrasonic SHM, which detects ultrasonic waves propagating a relatively long distance in a structure, is more effective for inside damage by analyzing the time of flight (ToF), attenuation, mode conversion, etc.

**Figure 1:**
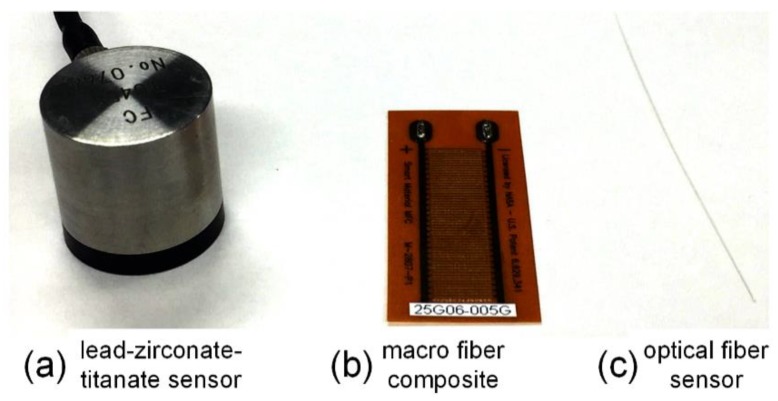
The typical ultrasonic sensors.

In ultrasonic SHM, the precise detection of the ultrasonic signal is the primary step, which can be accomplished by using high-performance sensors. The lead-zirconate-titanate (PZT) transducer is the most common device for both ultrasonic generation and ultrasonic detection [3,4]. By leveraging the well-established theory of piezoelectricity, PZT as a sensor can be designed and manufactured to possess high sensitivity to broadband frequency or resonant frequency. In addition, because the output of the PZT sensor is voltage, the ultrasonic signal can be easily recorded using an oscilloscope without the need for any additional demodulation system. Figure 1a shows a typical PZT transducer. Figure 1b shows another type of ultrasonic transducer, which is the macro fiber composite (MFC) invented by NASA in 1996 [5,6]. However, all these devices use piezoelectric ceramic as the active material, which has some drawbacks and will lead to some problems in certain application scenarios. For instance, because these devices are sensitive to electromagnetic interference, the detected ultrasonic strain signal cannot be distinguished clearly when the background electromagnetic noise is unneglectable. Furthermore, piezo sensors requiring both lead-in and lead-out wires do not have a multiplexing ability and their size is not suitable for being embedded into a material without the degradation of the material integrity.

The above-mentioned problems can be overcome if the sensor is replaced by an optical fiber sensor. Thus, many optical-fiber-based ultrasonic SHM techniques are being increasingly researched and utilized. Wild and Hinckley reviewed ultrasonic optical fiber sensors, including intensity-based sensors, interferometers, and fiber Bragg gratings (FBGs), and discussed their applications in acousto-ultrasonic detection [7]. Among these techniques, FBG sensors exhibit significant advantages, including their small size, light weight, immunity to electromagnetic interference, corrosion resistance, and high-temperature resistance. Some of these properties will be discussed in Section 2 and Section 6.

Since FBG was first manufactured in the late 1980s [8], it has been used in optical signal processing, communication, and sensing [9]. FBG can detect temperature and strain. It also can detect ultrasonic signals in liquids and solids [10,11,12] if the sensor head and the corresponding demodulation system are properly designed [13,14]. In addition, many practical issues should be comprehensively researched and carefully addressed for specific ultrasonic SHM techniques.

There are several FBG-based dynamic sensing solutions available on the market, as summarized in Table 1. I-MON 256 HS developed by Ibsen Photonics A/S has a 35-kHz bandwidth and 0.5-pm wavelength resolution [15]. The SmartFibres Inc. developed SmartScan with a scan frequency of 25 kHz for dynamic measurement [16]. Redondo Optics, Inc. developed a compact interrogator, FBG-Transceiver^TM^, which has a maximal sampling rate of 20 kHz per channel and a wavelength resolution of 5 pm at 100 Hz [17]. The three above-mentioned sensors can detect low-velocity impacts but is insufficient for high-frequency ultrasonic detection due to their limited maximal detectable frequencies. Redondo Optics, Inc. also released their FAESense^TM^, specific for acousto-ultrasonic detection. An ultrasonic bandwidth of hundreds of kilohertz and a strain resolution of 0.1 με/Hz has been reported [18]. I*Sense^®^ HS48M designed by Intelligent Fiber Optic System Corporation has a broad bandwidth up to 3 MHz [19]. Although FAESense^TM^ and I*Sense^®^ are applicable for acousto-ultrasonic detection, its performance to acoustic emission (AE) detection, a much more demanding and crucial application, is questionable. Therefore, it is timely and imperative to review the state-of-the-art techniques and to develop new techniques for FBG-based ultrasonic SHM.

This review summarizes the currently available FBG-based ultrasonic detection techniques and introduces their applications in ultrasonic SHM. The remainder of this review is organized as follows. In Section 2, the ultrasonic detection principle of normal FBGs and a specially designed FBG named the phase-shifted FBG (PSFBG) are introduced. In Section 3, two conventional and two cutting-edge demodulation techniques are presented and compared. In Section 4, the FBG sensor, under the influence of an ultrasonic Lamb wave, is discussed, followed by the discussion of sensor head designs and installation methods. In Section 5, typical and important studies on practical ultrasonic SHM including impact detection, acousto-ultrasonic detection, and AE detection, are referred to as examples. The structure of the paper, from sensor design to installation to utilization, is logical since it fits and covers all the actual procedures of ultrasonic SHM. Then, a brief discussion of future trends is conducted in Section 6. Finally, the conclusions are presented.

## 2. Ultrasonic Fiber Bragg Grating Sensor

### 2.1. Fiber Bragg Grating

**Figure 2:**
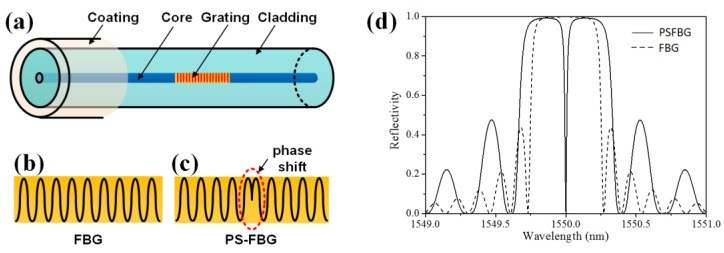
The introduction of fiber Bragg grating (FBG) and phase-shifted FBG (PSFBG). (**a**) The grating structure in an optical fiber; (**b**) the spatial index variation of an FBG; (**c**) the spatial index variation of a PSFBG; (**d**) the spectra of FBG and PSFBG.

Figure 2 shows that an FBG has a periodic grating structure at the core of an optical fiber, which is usually a single-mode fiber. The diameters of the core, cladding, and coating of the single-mode fiber are approximately 10, 125, and 150 μm. A small-diameter fiber with a diameter of 40–50 μm can also be used to replace the conventional single-mode fiber in FBG manufacturing [20]. The most important feature of the small diameter fiber is that it can be embedded in composite laminates without inducing strength deterioration. Another fiber that can be used in FBG manufacturing is the polymer optical fiber. FBG can be fabricated in a multimode micro-structured polymer fiber and used to monitor the hydrostatic pressure [21]. Marques et al. reviewed the FBG-based polymer optical fiber sensor and introduced its ultrasonic sensing capability when the sensor was in liquid. They claimed that the application of the sensor, for the safety of human life, is directly related with new solutions for ultrasonic SHM [22].

Figure 2b is a schematic diagram of the spatial index variation in an FBG written in a conventional single-mode silica fiber, described by Equation (1) [23].

(1) $$n_{eff}\left(z\right)=n_{eff0}+\Delta n\left[1+\cos\left(\frac{2\pi }{\Lambda_{0}}z+\phi \left(z\right)\right)\right]\;$$

where $n_{eff0}$ is the unperturbed effective refractive index, Δn is the maximum index change, $\Lambda_{0}$ is the grating period, and ϕ(z) is the grating chirp. Due to this periodic structure, FBG selectively reflects a part of the input broadband light and allows other light to pass through. The reflected (or the transmitted light) has a central wavelength called the Bragg wavelength, $\lambda_{B}$, which is expressed in Equation (2).

(2) $$\lambda_{B}=\;2n_{eff0}\Lambda_{0}\;$$

Although the reflection property of an FBG can be obtained by the direct-integration approach, straightforwardly solving it is difficult if the FBG has a complex spatial index variation profile or if the FBG suffers from non-uniform strain, such as ultrasonic waves. Thus, the transfer matrix method proposed is preferred because of its ease of implementation and high speed [24]. Assuming that the whole FBG is divided into multiple cascaded short segments with identical length, every section approximates to a uniform FBG, its light transfer property of which can be described by a transfer matrix. The complete mathematical expression of the whole FBG is obtained by multiplying all transfer matrices sequentially. By using this method, many specially designed FBGs can be successfully modeled [23]. The dotted line shown in Figure 2d is a simulated spectrum of a normal FBG.

When an FBG is stretched or compressed, or when it expands or shrinks, its Bragg wavelength shifts correspondingly. Equation (3) describes the relative Bragg wavelength shift Δ*λ_B_* for an applied strain ε along the fiber axis and a temperature change ΔT [8,13,25].

(3) $$\Delta \lambda_{B}=\;\left(C_{\varepsilon }\varepsilon +C_{T}\Delta T\right)\lambda_{B}\;$$

where $C_{\varepsilon }$ and $C_{T}$ are material constants. The typical values for Bragg wavelength shifts are approximately 1.2 pm/με for the strain sensitivity and 13.7 pm/K for the temperature sensitivity in the 1550-nm wavelength region [8].

### 2.2. The Principle of Ultrasonic Detection

According to Equation (3), the Bragg wavelength shift is linearly proportional to the applied static strain when the temperature is constant. However, the relation becomes complex when an FBG is under the influence of an ultrasonic Lamb wave in a plate-like structure. To simplify the analysis of the response of an FBG, the strain is assumed to be caused by a longitudinal ultrasonic wave with a sinusoidal form, which is expressed by Equation (4).

(4) $$\varepsilon (t)=\varepsilon_{m}\cos\left(\frac{2\pi }{\lambda_{S}}z-\omega_{S}t\right)\;$$

where $\varepsilon_{m}$ is the ultrasonic displacement amplitude, $\lambda_{S}$ is the ultrasonic wavelength, and $\omega_{S}$ is its angular frequency.

The ultrasonic strain changes the period and the index of an FBG [26]. Due to the geometric effect, the original position z in the grating is translated into a new position z′, which is expressed by Equation (5). Consequently, the new grating period of each segmented FBG in the transfer matrix method can be calculated.

(5) $$z^{\prime }=f(z,t)=z+\varepsilon_{m}\frac{\lambda_{S}}{2\pi }\sin\left(\frac{2\pi }{\lambda_{S}}z-\omega_{S}t\right)+\varepsilon_{m}\frac{\lambda_{S}}{2\pi }\sin\left(\omega_{S}t\right),z\in \left[0,L\right]\;$$

where L is the grating length. Additionally, the effective refractive index is changed due to the elasto-optic effect, as expressed by Equation (6).

(6) $$\begin{array}{ll} n_{eff}^{\prime }\left(z^{\prime },t\right) & =n_{eff0}-\Delta n\left(1+\upsilon \cos\left(\frac{2\pi }{\Lambda_{0}}f^{-1}\left(z^{\prime },t\right)\right)\right)\\ & -\left(\frac{n_{eff0}^{3}}{2}\right)\left[P_{12}-\upsilon \left(P_{11}+P_{12}\right)\right]\cdot \varepsilon_{m}\cos\left(\frac{2\pi }{\lambda_{S}}z^{\prime }-\omega_{S}t\right) \end{array}$$

where $P_{11}$ = 0.12 and $P_{12}$ = 0.275 are the stress-optic coefficients and υ = 0.17 is Poisson’s ratio. By substituting Equations (5) and (6) into Equation (2), the Bragg wavelength under the influence of an ultrasonic wave can be simulated.

Minardo et al. used the transfer matrix method to simulate the response of FBG to a longitudinal ultrasonic wave [26]. This research clarified that the FBG is barely sensitive to the ultrasonic wave when the ultrasonic wavelength is smaller than the grating length. Thus, the FBG sensor should be sufficiently short to fully receive all frequency information in a Lamb wave. The side-effect of the short uniform FBG is its gentle slope within its spectrum, correspondingly leading to a low detection sensitivity, which will be discussed in Section 3.2 and Section 3.3. In practical applications, when a uniform short FBG sensor is used to ensure sufficient bandwidth, averaging for noise reduction is usually necessary, albeit time-consuming. As a result, there is a conflict between the sensitivity and the bandwidth when a conventional FBG sensor is used for ultrasonic detection. The effect of the grating length in ultrasonic detection was also reported in other simulations and experiments [27,28,29].

### 2.3. Special Fiber Bragg Gratings

A PSFBG, which is a special type of FBG, has unique characteristics in ultrasonic detection. Its manufacturing process is similar to that of normal FBG, except that a π phase-shift is inserted in the middle of the grating area, as shown in Figure 2c [30]. The two sections of the uniform FBGs that are next to the phase-shift area constitute a Fabry–Perot structure. Therefore, the spectrum of the PSFBG differs from that of the normal FBG dramatically, showing a narrow valley in the middle of the reflected spectrum, i.e., a narrow peak in the middle of the transmitted spectrum. To simulate the spectrum of a PSFBG, a π phase shift matrix should be added into a series of transfer matrices.

The solid line shown in Figure 2d is the spectrum of a PSFBG that has the same grating length, grating period and refractive index as the uniform FBG. The slope in the valley of the PSFBG spectrum is steep and the full width at half maximum (FWHM) of the valley is narrow. These characteristics are beneficial for not only static measurement [31,32], but also for ultrasonic detection [33]. First, the ultrasonic sensitivity can be better than that of a uniform FBG due to its steep slope at the central peak (or valley) of the spectrum. Furthermore, a PSFBG confines its light field to the phase-shifted area and its effective length could be very short, thereby allowing it to detect ultrasonic signals up to the megahertz range [34].

**Figure 3:**
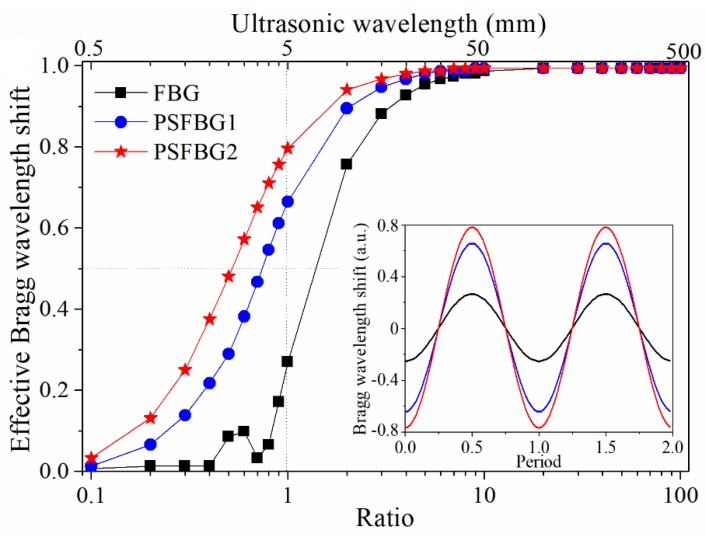
The responses of FBG and PSFBGs to longitudinal waves with different ultrasonic wavelengths. The inset shows the time-domain Bragg wavelength shifts of FBG and PSFBG.

Figure 3 compares the simulated responses of FBG and PSFBGs to longitudinal waves with different ultrasonic wavelengths. All gratings have the same length (5 mm) and an original wavelength of 1550 nm. PSFBG2 has a higher index modulation compared to PSFBG1. The vertical axis of Figure 3 represents the effective Bragg wavelength shift, which is the ratio of the Bragg wavelength shift under ultrasonic stimulation to the Bragg wavelength shift under static loading, and the horizontal axis represents the ratio of ultrasonic wavelength to grating length. All curves can be divided into two parts. When the ratio is larger than 10, all FBGs have almost the same effective Bragg wavelength shift, indicating that the ultrasonic strain affects all gratings with the coefficient approaching $C_{\varepsilon }$. However, when the ratio reduces from 10, all the effective Bragg wavelength shifts become smaller. The curve of normal FBG decreases most rapidly, while the curve of PSFBG2 decreases relatively gently. In the inset of Figure 3, the time-domain Bragg wavelength shifts of FBG and PSFBGs also clearly exhibit their different responses. Provided that the speed of the ultrasonic wave is 5 km/s, the 3-dB bandwidth of FBG, PSFBG1, and PSFBG2 are 0.71, 1.35, and 1.92 MHz. This means the bandwidth of PSFBG2 is about 2.7 times broader than that of normal FBG. In light of the above analyses, the PSFBG provides a very feasible way of resolving the conflict between ultrasonic bandwidth and sensitivity, and its performance can be further improved by using a larger index variation in order to more tightly confine the light field to the phase-shifted area.

In addition to PSFBG, multiple-phase-shifted FBG was designed by Azmi et al. The FBG sensor with 17 phase-shifted areas attained a sensitivity enhancement of nearly 20 dB compared to a standard FBG and achieved a bandwidth in the kilohertz range [35]. However, the fabrication of this sensor is difficult, and the quality of the detected signal has not been presented.

## 3. Demodulation Techniques

### 3.1. Comparison of Demodulation

When an FBG is used to detect static strain, its Bragg wavelength can be tracked using instruments such as the optical spectrum analyzer. Because detecting ultrasonic strain requires a sub-micron-strain resolution [36] and an over-kilohertz bandwidth [37,38], tracking the Bragg wavelength is usually not feasible due to the limitations of the optical spectrum analyzer in terms of its sensitivity and demodulation speed. To meet the requirements of ultrasonic detection, demodulation technique is required.

The demodulation techniques can be divided into four categories according to the light source that is used, as summarized in Table 2. The first and second techniques use a broadband light source and a laser light source, respectively. They are also called power detection and edge filter detection [7]. In addition to these two mainstream and relatively mature techniques, the other two cutting-edge demodulation techniques are in the laboratory stage. The light sources used in these two techniques are erbium-doped fiber laser (EDFL) and modulated laser. These two designs improve the sensing performance in other important aspects, such as the multiplexing ability and resistance to environmental disturbances, although the sensitivity and bandwidth of an ultrasonic sensing system are still of uppermost priority.

### 3.2. Broadband Light Source

**Figure 4:**
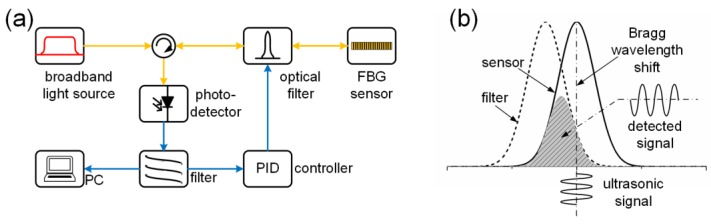
The FBG demodulation systems using a broadband light source. (**a**) The schematic diagram of the system setup; (**b**) the detection principle.

**Figure 5:**
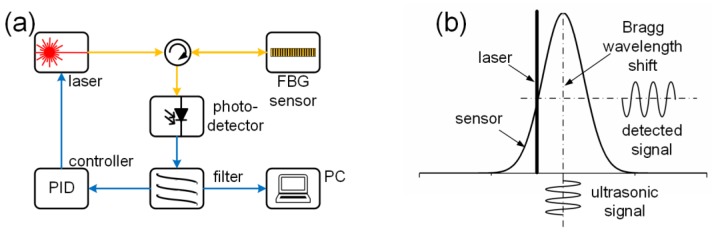
The FBG demodulation systems using a laser light source. (**a**) The schematic diagram of the system setup; (**b**) the detection principle.

**Figure 6:**
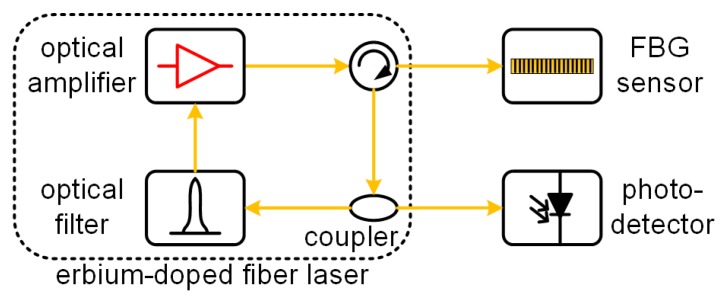
The FBG demodulation system using an erbium-doped fiber laser.

**Figure 7:**
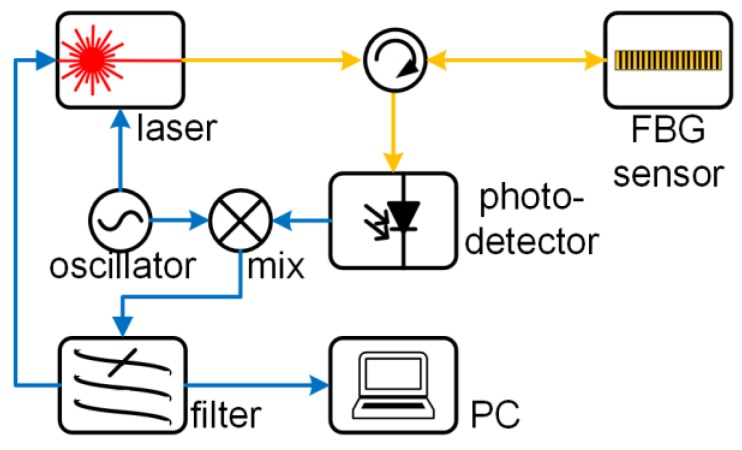
The FBG demodulation systems using modulated.

Figure 4a,b show the system configuration and the detection principle of the first demodulation technique. The yellow line and blue line represent the pathway of the light and electrical signals, respectively. This notation is also used in Figure 5, Figure 6 and Figure 7. A broadband light source, such as an amplified spontaneous emission or a super-luminescent diode, has spectral power density S(λ). The light source, after being tailored by an optical filter, illuminates the FBG sensor. The spectra of the filter $F_{filter}\left(\lambda \right)$ and sensor $F_{sensor}\left(\lambda \right)$, which can be either transmission or reflection depending on the system configuration, have an overlapped area. The photodetector with response factor $R_{D}\left(\lambda \right)$, which is the product of the responsivity and resistance, integrates all the power over its full responsible wavelength range. If the strain shifts the Bragg wavelength $\Delta \lambda_{B}$, the final output voltage of the system V will also change as a function of $\Delta \lambda_{B}$, given by Equation (7) [39,40].

(7) $$V\left(\Delta \lambda_{B}\right)=\int R_{D}\left(\lambda \right)S\left(\lambda \right)F_{filter}\left(\lambda \right)F_{sensor}\left(\lambda -\Delta \lambda_{B}\right)d\lambda \;$$

Since the output light power from a broadband light source and the response of a photodetector are uniform in a certain wavelength range, S(λ) and $R_{D}\left(\lambda \right)$ can be treated as constants. Assuming the wavelength of the sensor is longer than that of the filter, the spectra of the two FBGs go from overlap to separation for a linearly increasing strain. As a result, a decrease in the output power will be observed. Although the curve of $V\left(\Delta \lambda_{B}\right)$ is not absolutely linear, a quasi-linear area can be found over a small wavelength shift range and be used to represent the waveform since the strain induced by the ultrasonic source, such as AE, is usually in the sub-micron strain range [36]. Based on this detection principle, various sensing techniques can be implemented by combining different filters and sensors.

Perez et al. proposed a matched filter interrogation system that used a pair of FBGs with marginally different Bragg wavelengths and applied this system to successfully detect a signal generated by the PZT transducer that was directly bonded to the FBG sensor [41,42]. This may be the first attempt at ultrasonic detection using the first demodulation technique. Both numerical and experimental results showed that for maximizing the sensitivity, this system exhibits an optimum Bragg wavelength difference between these two FBGs, the value of which is noise-source-dependent [39]. To ensure the best performance, a feedback controller for tuning the optical filter is always necessary, as shown in Figure 4 [43].

The matched FBG filter can be replaced by another filter, such as a microelectromechanical system [44], a photorefractive crystal [45], an FBG with narrow FWHM [46], or a Mach–Zehnder interferometer [47]. The optical filter can also be an arrayed waveguide grating, which was proposed by Sano and Yoshino [48]. This system can be further developed by utilizing an FBG sensor with a 1-mm grating length. Then, a Lamb wave can be successfully detected [49,50]. Theoretical analyses of the detection sensitivity of this system can be found in References [48,51]. Using a sensor and/or filter with a steeper slope will cause a larger output voltage difference under the same wavelength shift and, thus, is beneficial to the system sensitivity. For example, two cascaded PSFBGs can effectively improve the ultrasonic sensitivity [52]. PSFBG can also be demodulated by interferometric interrogation with a very steep slope [53].

This demodulation method has a multiplexing potential because of the used broadband light source, e.g., an amplified spontaneous emission with a typical wavelength of 1530–1570 nm. However, even when using PSFBG, the sensitivity is just enough to real-time detect the Lamb wave generated from an MFC driven by a signal with a 150 peak-to-peak voltage [40]. The relatively low sensitivity, which is manifested as a low signal-to-noise ratio due to the physical nature of the broadband light source, restricts its application area.

### 3.3. Laser Light Source

Figure 5a,b show the system configuration and detection principle of the second demodulation technique, which is based on a laser light source such as the tunable semiconductor laser. Although a recent study proposed a technique based on detecting the sidelobe reflectivity, which will lead to a weak optical single [54], a conventional system adjusts the wavelength of the laser on the main spectral slope of an FBG sensor. By using a photodetector to receive the reflected or transmitted light power of the FBG, the ultrasonic signal can be represented as a voltage fluctuation. Because the grating slope in a short range is linear, the detected signal also truly reflects the ultrasonic signal. The AC components of the final voltage signal can be expressed by Equation (8) [55].

(8) $$V_{S}=\Delta \lambda_{B}GR_{D}P\;$$

where $V_{S}$ is the detected AC signal voltage, $\Delta \lambda_{B}$ is the Bragg wavelength shift caused by strain, G is the grating slope, $R_{D}$ is the photodetector’s response factor, and P is the input laser power. Because the output voltage is proportional to the grating slope and input laser power, using an FBG with a steep slope and a light source with a high power benefits the amplitude of the detected signal. As the noise level limits the sensitivity of a system, considerable efforts in this demodulation technique are made to optimize the signal-to-noise ratio.

The signal amplitude can be amplified by using a transmit-reflect detection method [56]. The reflected and transmitted lights are detected by separated photodetectors and the resulting signals are subtracted from each other for signal amplification. However, this method cannot reduce the noise. Wu and Okabe used PSFBG to improve the amplitude of the detected signal and they also used a balanced photodetector to simultaneously detect the reflected and transmitted light to eliminate the laser power noise, which is the dominant noise source in their system. These two updates resulted in a 9 nε/Hz^−1/2^ sensitivity [57].

Hu et al. used another reference sensor to reduce the frequency noise additively to achieve up to a 20-dB sensitivity improvement [58]. Another research article claimed that the shot noise also limits the detection accuracy [55]. In addition to laser power noise, frequency noise, and shot noise, there are other noise sources [59]. Although a specific sensing system needs targeted and comprehensive analyses to figure out the dominant noise source, the second demodulation technique is always more sensitive to ultrasonic waves than the first demodulation technique.

Instead of a semiconductor laser, a fiber laser with its wavelength tuned through a tunable filter can also illuminate the FBG sensor [60]. The fiber laser and FBG are connected through an optical isolator to prevent laser instability induced by the FBG-reflected light.

### 3.4. Erbium-Doped Fiber Laser

Recently, FBG demodulated by an integrated EDFL was proposed. Figure 6 shows its typical configuration. Unlike the system that isolates the FBG and fiber laser via an isolator [60], the FBG sensor and the EDFL are integrated and will interact in this technique. The reflected light from the FBG sensor reenters the laser ring cavity via a circulator. Thus, the detected signal from the optical coupler is affected by not only the ultrasound-encoded FBG but also by the dynamic properties of the EDFL [61], which is its relaxation oscillation. When the ultrasonic frequency approaches the frequency of the relaxation oscillation, the detected signal can be amplified significantly albeit with distortion. The other main characteristic of this design is its versatility. Especially the increased flexibility in the EDFL endows the system with an automatic tuning ability and consequently, a resistance to disturbance.

Tsuda first reported a vibration sensing system by simply integrating an FBG sensor with an EDFL, and demonstrated its environmental resistance. The detection principle was explained as the uneven gain spectrum of the erbium-doped fiber [62]. Han et al. established a system by inserting a tunable Fabry–Perot filter into the laser ring cavity. As the wavelength of the filter is controlled and stabilized on the slope of the FBG sensor by an external electrical signal [63], this system does not have an automatic tuning ability. Then, two additional studies were conducted. One is the multiplexing ability achieved by cascading multiple ring cavities and the Fabry–Perot filters, though the complexity and cost of the system increased [64]. The other is the resistance to environmental disturbances by using a tandem FBG to replace the Fabry–Perot filter and gluing it at the same position as that of the FBG sensor. This design was demonstrated for the detection of an ultrasonic tone-burst signal centered at 200 kHz over a static strain range of 1800 με [65]. Another experiment using a similar setup was demonstrated for ultrasonic detection over a temperature range of 25–85 Celsius [66]. The PSFBG can also be used in this demodulated technique. Because the FWHM of the PSFBG is very narrow, the wavelength of the EDFL is determined solely by the PSFBG after its central peak area is extracted by another filter [67]. Multiple FBG-EDFL structures were studied and compared in Reference [66]

A numerical model was constructed for revealing the relationship between the signal enhancement and the frequency of the relaxation oscillation and investigating the influences from the parameters of the EDFL [66,68]. Since the frequency of the relaxation oscillation was predetermined once a system was set up, a deconvolution data processing method could be utilized to reconstruct the actual ultrasonic waveform [69].

In the first and second demodulation techniques, the maximal Bragg wavelength shift usually cannot be larger than half of the FWHM of the sensor and filter. However, this condition becomes difficult to satisfy because of the environmental disturbances from the temperature and static strain in a practical SHM. Using a feedback controller is the most straightforward method for enhancing the robustness and expanding the service range of a sensing system [70]. However, a feedback controller requires extra electrical devices and its performance is largely dependent on the control algorithm. Therefore, the auto-tuning accomplished by the third demodulation technique is its great advantage.

### 3.5. Modulated Laser

In contrast to the detection of the power fluctuation introduced in Section 3.2, Section 3.3 and Section 3.4, there are other methods. One method is the Pound–Drever–Hall (PDH) technique, the original purpose of which was to stabilize the laser wavelength. As shown in Figure 7, the light path of this design is similar to that in the second demodulation technique, except that the laser light source is modulated by an electrical signal with a frequency over a few hundred megahertz. The wavelength of the laser vibrates around the FBG’s Bragg wavelength rather than locking onto its spectral slope. After the reflected light from the FBG sensor is detected by a photodetector, the electrical signal encoded with the ultrasonic signal is mixed with the electrical modulation signal. The error signal from the mix is the ultrasonic signal, while the low-frequency vibration is used to tune the laser wavelength.

The ultrasonic sensitivity achieved by the PDH technique reaches 5 pεHz^–1/2^ for frequencies larger than 100 kHz when the sensor is PSFBG. This sensitivity is higher than those of previously reported techniques because both the laser power noise and frequency noise were reduced [71]. It has been demonstrated that the PDH technique can demodulate the Bragg wavelength shift caused by an ultrasonic signal with a frequency of around 200 kHz in liquids [72] or solids [73].

In addition to the PDH technique, a two-step modulation technique consisting of both amplitude modulation and frequency modulation was proposed recently [74]. The ultrasonic signal with a central frequency of 200 kHz was read directly from the electrical error signal when the amplitude modulation was cancelled.

## 4. The Ultrasonic Wave from Structure to Fiber

### 4.1. FBG under the Influence of a Lamb Wave

In a plate-like structure, the propagating wave confined by the thickness of the plate is usually a Lamb wave, which is one type of guided waves [75,76,77]. Its long propagation distance is beneficial to ultrasonic SHM; however, its dispersion and complex mode properties of both symmetric modes (S modes) and antisymmetric modes (A modes) are not advantageous to the following wave interpretation. Because researchers are keen to possess all the necessary information of the wave, researching the FBG under the influence of an ultrasonic Lamb wave is important. In practice, this is about the design and optimization of the FBG installation method, which has a large impact on the sensing properties, including sensitivity, bandwidth, resistance to environmental disturbance, etc.

**Figure 8:**
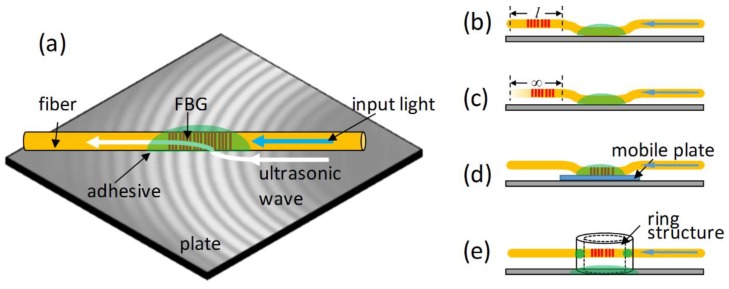
The FBG under the influence of an ultrasonic wave. (**a**) surface-mounted; (**b**) cantilever structure; (**c**) remote sensing; (**d**) mobile sensor; (**e**) piezo ring structure.

An FBG sensor can be embedded into a target material, such as a composite material, to detect damage [78]. However, this embedding is a sophisticated technique and may lead to other side effects, such as birefringence and non-uniform-strain-induced optical spectrum distortion [79]. Thus, a common fiber installation approach in ultrasonic SHM is to mount the sensor onto the structural surface by using epoxy, cyanoacrylate, or some other adhesives, as shown in Figure 8a. The ultrasonic sensitivity of the FBG can be improved by carefully choosing the adhesive materials and fiber coating materials, as both of them can influence the strain transfer from the structural plate to the optical fiber [80,81,82,83].

Unlike a PZT sensor that has a round shape and exhibits omni-directional sensitivity, an FBG has a slim cylindrical structure, exhibiting a specific response to the ultrasonic wave. First, FBG is directionally sensitive [27,42,84]. It is mainly sensitive to ultrasonic waves that propagate along the fiber direction, but not to those that propagate in its transverse direction. Its response decreases following a cos function when the angle between the FBG and ultrasonic source increases. The sensitivity distribution of a PSFBG is similar to that of an FBG when the distance between the sensor and the ultrasonic source is large enough, but becomes complex when the distance is shorter than 4 cm in the experimental setup of the research [85]. Due to this directional sensitivity, FBG was believed to be not suitable for the detection of a shear wave, the motion of which is perpendicular to the direction of wave propagation [86]. However, recently, Harish et al. have experimentally demonstrated that the FBGs, when placed along a direction perpendicular to a propagating elastic wave, can detect the fundamental shear horizontal mode [87]. Second, an FBG is sensitive to the in-plane motion rather than the out-of-plane motion [88]. The in-plane motion that is transferred from the plate to the fiber can be effectively received by FBG within an optical fiber. The advantage of this property is that the signal detected by FBG is purely derived from the in-plane elastic strain. However, due to these unique properties of FBG, the signals detected by FBG and PZT usually have different waveforms, leading to difficulty in comparison.

### 4.2. Ultrasonic Wave Propagation along the Fiber

The optical fiber is not only an ideal optical waveguide but also an ideal ultrasonic waveguide as a high-frequency ultrasonic wave can propagate along the fiber with low attenuation. The expected propagation distance reaches tens of meters [89]. If an ultrasonic wave is guided by an optical fiber and then detected by a conventional piezo sensor, the leakage of a pipeline can be evaluated according to additional ultrasonic attenuation [90], and local structural hot spots can be monitored to extend the temperature limitations of PZT [91].

Figure 8b shows the cantilever structure. The FBG sensor is not directly mounted on the target structure, but is glued to a position near the grating area. As a result, the grating within a short length of the optical fiber is suspended in midair. An ultrasonic wave enters the fiber via the adhesive point, propagates along the fiber, and forms a standing wave between the adhesive point and the free end of the suspended fiber. Thus, the FBG sensor within this structure is very sensitive to a few frequency components that are determined by the length of the suspended fiber. In other words, this cantilever structure converts the FBG from an ultrasonic broadband sensor into a resonant sensor [92,93]. Moreover, the FBG cantilever structure is free from static strain, and multiple sensors with identical wavelengths can be powered by a single laser to achieve multiplexing detection [94]. As an alternative to the cantilever structure, a bridge structure was designed when two positions near the FBG were both glued to a target structure [95]. As a result, it shows very similar properties to the cantilever structure.

**Figure 9:**
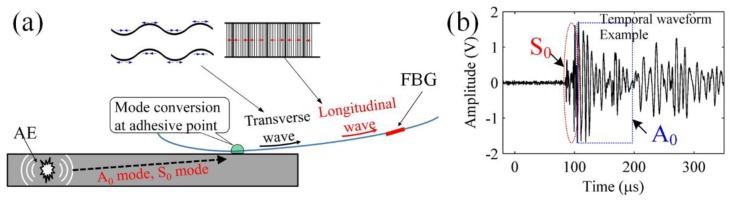
The ultrasonic wave propagation along an optical fiber. (**a**) The principle of remote sensing; (**b**) a typical waveform detected by remote sensing.

Figure 8c shows the remote sensing structure. Unlike the cantilever structure with a short suspended fiber, the fiber length in the remote sensing structure is literally infinite. As shown in Figure 9a, a fundamental longitudinal wave with an axial strain component and a fundamental flexural wave with mainly a shear strain component appear in the optical fiber. Then, the FBG sensor selectively receives the longitudinal wave because of its sensitivity to the axial strain. In addition, the longitudinal mode shows no dispersion in the optical fiber due to the longer ultrasonic wavelengths compared to the diameter of the optical fiber. It was verified that the optical-fiber-based waveguide allows the FBG sensor to respond to the original ultrasonic waveform at the adhesive point [96]. Figure 9b exhibits a typical waveform detected by the remote sensing structure, showing the S_0_ and A_0_ modes clearly.

Wee et al. researched the sensitivity of the remote sensing structure by considering the wave attenuation [97] and then indicated that ultrasonic waves can propagate both forward and backward along an optical fiber [98]. Furthermore, Yu et al. performed simulations to clarify how the adhesive length in the remote sensing configuration influenced ultrasonic detection [99].

### 4.3. Mechanically Aided Sensor

Integrating FBG with mechanical devices can substantially change its properties to ultrasonic waves and further exploit its sensing potential. One type of mechanically aided FBG sensor head is the mobile sensor. An FBG is mounted on the surface of a small acrylate plate with a 1.6 mm thickness rather than directly onto the target structure [100]. Then, this sensor head is attached to the target structure by a couplant, as shown in Figure 8d. This sensor head is mobile and insensitive to static strain because the viscous couplant impedes the strain transfer from the structure to the acrylate plate. However, the mobile sensor is still effective in ultrasonic detection albeit with additional attenuation from the acrylate plate [36].

Another mechanically aided FBG sensor head is the piezo-optical ring sensor. An FBG that acts as the sensor media was bonded across a metal ring structure, as shown in Figure 8e. The metal ring picks the vertical motion from the target structure and then enters its resonant frequency, which is determined by its geometric parameters. Subsequently, the FBG sensor is stretched and compressed by the vibration of the ring. According to this detection principle, this sensor has three main and unique properties [88,101]. First, it is a resonant sensor. Second, it is sensitive to the out-of-plane strain. Third, it has omni-directional sensitivity owing to the round shape of the metal ring.

## 5. FBG in Ultrasonic Structural Health Monitoring

### 5.1. Comparison of Ultrasonic SHM Techniques

Impact detection, acousto-ultrasonic detection, and AE detection are three typical ultrasonic SHM techniques. The first technique detects the Lamb wave caused by an impact. A well-designed sensor network can estimate the impact location, impact energy, and damage level by evaluating the ToF and ultrasonic attenuation, etc. [102]. The acousto-ultrasonic detection receives the Lamb wave generated by an actuator under deliberate control [103]. Because the wave is repeatable, the damage in a material can be evaluated more precisely and even quantitatively by comparing the amplitude attenuations or by examining the mode change of the waveform. AE detection collects the ultrasonic signals that are spontaneously emitted from a defect at the moment of its occurrence [37,38]. It is a powerful and qualitative SHM technique achieved by analyzing the cumulative hits, energy distribution, peak frequency, etc. Impact detection and AE detection are passive approaches and acousto-ultrasonic detection is an active approach because an ultrasonic actuator is needed.

**Figure 10:**
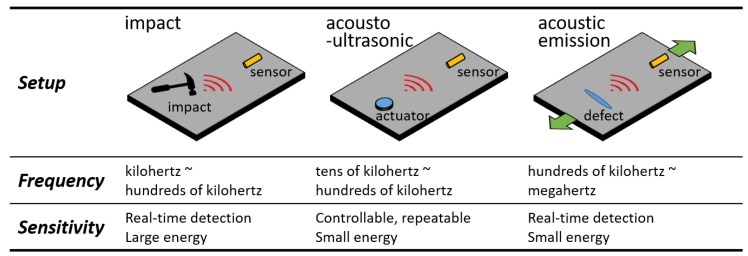
The comparison of three types of ultrasonic structural health monitoring techniques.

Figure 10 compares these techniques and shows their frequency and sensitivity properties. The Lamb wave in impact detection has the largest energy and lowest frequency. However, real-time detection should be performed because averaging for noise reduction cannot be carried out. The acousto-ultrasonic detection requires a higher frequency range. Although the magnitude of the ultrasonic signal from the actuator is small, averaging owing to the controllability of the input signal is widely acceptable, albeit with a higher time cost and lower efficiency. The required frequency of AE detection may reach megahertz. Furthermore, real-time detection is needed since the AE signals cannot be repeated. The above comparisons conclude that impact detection is easy and can be accomplished by many FBG-based systems; while AE detection is challenging and can only be realized by a few systems. Table 3 summarizes and correlates the reviewed papers and the techniques in FBG sensor design, demodulation, installation, and applications.

Although ultrasonic SHM can be used to monitor the health statuses of various materials, many studies were conducted on the SHM of composites, especially the carbon fiber reinforced plastics (CFRPs) that can be used in aerospace industry. Because CFRP is an anisotropic material and can have a complex laminated structure, it is difficult to detect and discriminate between multiple damage types, including matrix cracks, delaminations, and fiber breakages. The following introduced applications of FBG in ultrasonic SHM mainly focus on composite materials.

### 5.2. Impact Detection

Conventional uniform FBG is suitable for impact detection because the Bragg wavelength shift induced by an impact is sufficiently large. Research indicates that a low-energy impact can induce a maximum strain level of up to 1000 με, approximately corresponding to a 1.2 nm Bragg wavelength shift [104]. In contrast, PSFBG with a narrow FWHM will distort the detected signal and is therefore not suitable for impact detection. Vella et al. used a high-speed microelectromechanical filter to fully represent the FBG spectrum change with a sampling frequency of 100 kHz. Their findings showed that the evolution of the FBG spectrum in response to an impact was much more complicated than a simple Bragg wavelength shift [44]. Goutham et al. used a photorefractive crystal and adopted an FBG sensor pair configuration to overcome the FBG’s problem of directional sensitivity and subsequently achieved impact location identification [45]. Lee et al. used the FBG mobile sensor to detect impact waves and demonstrated that the sensor, together with a PZT transmitter, can be extended to acousto-ultrasonic detection to evaluate the length of the impact-induced delamination [28].

Impacts on various structures have been monitored by using FBG. Gomez et al. used an FBG network to detect the impact location and energy on an aeronautical composite and emphasized the importance of interrogation speed, with a recommended value of several hundreds of kilohertz [105]. Tsutsui et al. detected the impact damage on stiffened composite panels using embedded FBGs that were written on small-diameter optical fibers and discussed the relationships among the optical response, the impact load, and the impact damage [106]. Shin et al. used an FBG array to detect the impact on a wind turbine blade and evaluated the severity of the impact [107]. Impact-induced delamination onset in composite laminates can also be analyzed after decomposing the detected low-velocity impact signal by wavelet transform [108].

**Figure 11:**
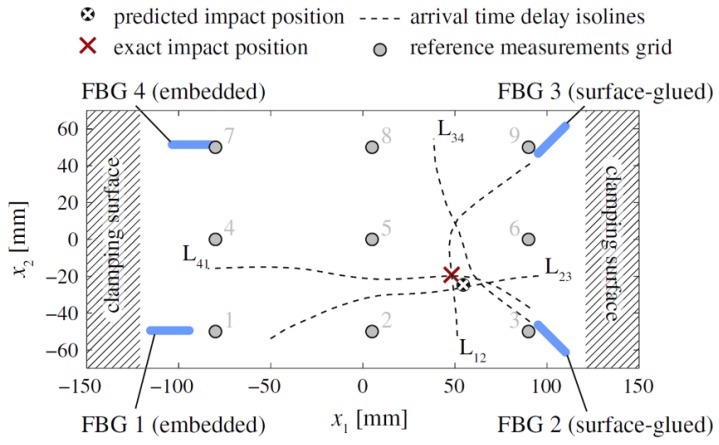
The isolines of equal arrival time delays and predicted impact location [104].

When the frequency is lower than 100 kHz in the impact detection, it is considered the A_0_ and S_0_ modes of the Lamb wave are approximately non-dispersive. Thus, the ToFs could be different when multiple sensors receive either the A_0_ or S_0_ mode resulting from the same impact. The ToFs are calculated using the arrival times of the waves at the sensors, usually determined using the threshold method. The conventional triangulation algorithm can identify the impact position on a 2D plane if the material is isotropic and the sensor is omni-directional [109]. However, this task becomes complex and requires advanced data processing techniques when using FBGs with highly directional sensitivity and monitoring an anisotropic plate such as a composite laminate. Frieden et al. used four FBGs to obtain the arrival times of the A_0_ mode of Lamb waves and proposed the interpolation of a known data set to identify the impact location [104]. They demonstrated their data process method on a CFRP laminate with a dimension of 300 × 140 mm. As shown in Figure 11, two FBGs were embedded and two FBG were surface glued. First, the hammer impact was performed on a grid of nine points (gray dots) to provide references for the interpolation-based localization method. Then, the plate was impacted at the position (50 mm, −20 mm) with an energy of 3.4 J. The isolines L_12_, L_23_, L_34_, and L_41_ are the lines where the interpolated response surfaces of the four different couples of sensors take the values of the arrival time. From this data, the intersections of the isoline pairs (L_12_, L_23_), (L_23_, L_34_), (L_34_, L_41_), and (L_41_, L_12_) were calculated and the predicted impact location, which was the average of the intersections, was close to the exact location with a 5.5-mm distance error.

There are other research articles on position identification using Lamb waves detected by FBG. Kirkby et al. used an optimization scheme that minimizes an error function to identify impact events within an error of a few centimeters [84]. Jang et al. adopted neural networks and wavelet transform to precisely evaluate the impact location on a composite aero-vehicle structure [110]. Lu et al. used wavelet transform incorporated with a model of training of least squares support vector machines to achieve a 3.7-mm average impact localization error in a 300 × 300 mm composite plate [111]. Shrestha et al. used a 1D-array FBG sensor configuration to identify the impact location on a composite wing and demonstrated that the result was comparable to that from a 2D sensor network configuration. Furthermore, they demonstrated that both the root-mean-squared-based and correlation-based algorithms can localize the impact well [112]. Kim et al. used a normalized cross-correlation method to localize the low-velocity impact in a stiffened composite panel. The average error of their method using four FBG sensors was 14.23 mm [113].

### 5.3. Acousto-Ultrasonic Detection

FBG can be used in acousto-ultrasonic detection to sense an ultrasonic Lamb wave that propagates in an isotropic Perspex plate [25], isotropic metal plate [29], bent plate [114], anisotropic CFRP laminate [115], or glass fiber reinforced plastic laminate [116]. The FBG sensor can be demodulated by a broadband light source or a laser light source [117]. When a broadband light source is used, FBG sensor can also be demodulated by a broad-bandwidth filter and a narrow-bandwidth filter for the detection of both the strain and the ultrasonic signal [118]. Moreover, most evaluations and calibrations of the FBG ultrasonic sensors were conducted based on the acousto-ultrasonic configuration because of the controllability and repeatability of the input ultrasonic wave, as indicated by many examples in Section 3.

**Figure 12:**
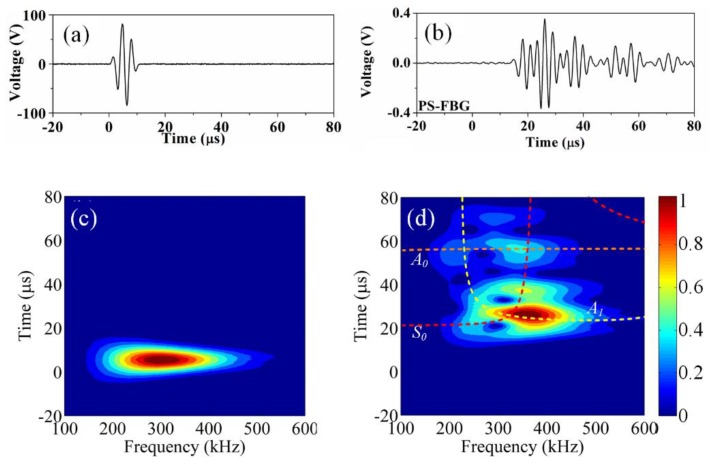
The typical waveform in acousto-ultrasonic detection. (**a**) input signals; (**b**) detected signals; (**c**,**d**) corresponding wavelet transform results.

Figure 12 shows typical experimental results of acousto-ultrasonic detection using an FBG on a composite laminate. The used CFRP with a structure of [45/0/−45/90]_3s_ has a dimension of 220 × 220 × 3.4 mm^3^ (L × W × H). An ultrasonic signal with a peak-to-peak voltage of 150 V drove a 6-mm MFC to generate a Lamb wave. A distance of 70 mm away from the MFC, an FBG was glued along the central line to receive the ultrasonic signal. Figure 12a,b show the input and the detected signals, respectively. Once damage occurs in a composite, the detected Lamb wave will correspondingly change. Takeda et al. used a small-diameter FBG to detect the signal and to quantitatively evaluate the debonding process in a CFRP double-lap type coupon specimen according to the arrival time delay of the wave [27]. The same method was also used for a skin/stringer structure of an airplane, and a new damage index was proposed to evaluate the size of the debonded area [119]. Figure 12c,d are the corresponding wavelet transform results of wave in Figure 12a,b. By comparing to the theoretical curves of the group velocities of this CFRP quasi-isotropic laminate, the A_0_, A_1_, and S_0_ modes of the lamb wave can be determined. After the mode discrimination, a more precise analysis can be conducted. The modes of the Lamb wave will be converted and reflected in the change of the detected ultrasonic signal because the debonding and delamination in a laminated composite will cause the plate thickness to vary locally. Okabe et al. investigated the mode conversion phenomenon on laminates with artificial delamination and used the FBG acousto-ultrasonic detection system to evaluate the delamination length [120].

Acousto-ultrasonic detection has similar practical problems to impact detection. Betz et al. used an FBG rosette that comprised of three FBGs aligned in a triangle to solve the problem of directionally dependent sensitivity. After using the genetic algorithm to calculate that intersection and account for any ambiguities from the Lamb wave measurements, the detected location was close to the actual damage position [121]. Wu proposed and demonstrated a distributed hybrid PZT/FBG sensor network for evaluating debonding in composites. A damage index, which was developed for extracting features in sensor signals that were related to damage in the structure, was calculated individually for each actuator–sensor path to represent the health status of the area associated with the path [122].

Like uniform FBG, PSFBG achieves a satisfactory performance in practical acousto-ultrasonic detection. Wu et al. cascaded two PSFBGs to achieve real-time ultrasonic detection [40]. Hudson et al. demodulated the PSFBG by a laser and to real-time monitor the cure process of composites [123]. Guo and Yang detected ultrasonic wave using PSFBG demodulated by a PDH system and successfully discriminated between the A and S modes [73].

### 5.4. Acoustic Emission Detection

A few research articles that used normal FBGs to detect AE signals were found because of the obstacles from the low energy and high frequency of the AE signals [38,124]. Baldwin and Vizzini detected pencil lead breakages, metal-to-metal impacts, and AE signals that were generated in a composite specimen [125]. Mabry et al. also detected AE signals in a tensile test of CFRP [126]. Due to the low sensitivity of their systems, much fewer AE hits were detected by the FBG than by the conventional PZT sensor. The detected AE hits possibly corresponded to severe defects, such as delamination and fiber breakages. Tsuda et al. used an FBG cantilever structure to detect AE signals during a pressure test of a CFRP tank [127]. However, further signal analysis was difficult owing to the resonance property of the sensor head.

**Figure 13:**
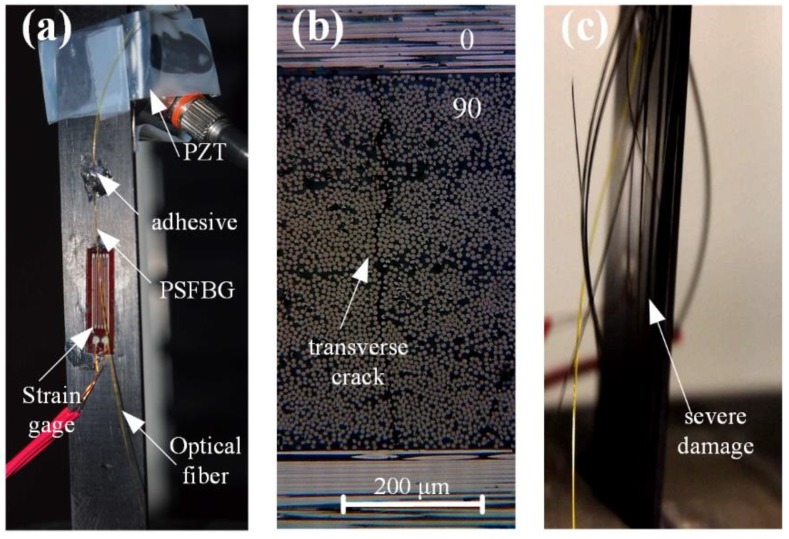
The acoustic emission detection in a tensile test. (**a**) the setup of AE detection using PSFBG; (**b**) transverse crack; (**c**) severe damage.

**Figure 14:**
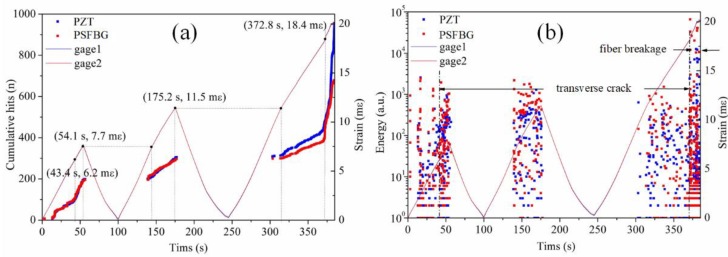
The statistical analyses of the detected AE signals. (**a**) Cumulative AE hits; (**b**) energy distribution.

AE detection using FBG has become more realistic after the emergence of the PSFBG-based ultrasonic sensor. Raju et al. used PSFBG that was demodulated by a tunable laser to detect AE signals for failure characterization in composite top-hat stiffness. Their results were in good agreement with those of the conventional PZT sensor although still fewer AE signals were obtained [128]. Wu et al. used their PSFBG system that had a higher sensitivity and collected multiple AE signals [129]. Figure 13a shows the experimental setup. On the CFRP laminate with a structure of [0_2_/90_2_]_s_, the optical fiber sensor, PZT, and strain gages were mounted. During a loading–unloading tensile test, both transverse cracks and severe damage were observed, as shown in Figure 13b,c. Figure 14 shows the statistical analyses of the AE signals detected by both PZT and PSFBG [130,131]. Correlating the AE signal to the strain curve manifests that the AE signals mainly appeared when the strain exceeded the previous maximal strain level. The accumulative AE hits detected by the PSFBG sensor were comparable to those detected by the conventional PZT sensor, as shown in Figure 14a. At 370 s, a clear knee point in Figure 14a and a wider energy distribution in Figure 14b separate the signals into two categories. The signals generated before and after a strain of 0.0184 may correspond to the transverse cracks and fiber breakages, respectively. Then, another AE detection experiment in a three-point bending test was also conducted using the same sensing system. Only the AE signal from the matrix transverse crack was generated and precisely detected. Because the AE signal from transverse crack has the smallest energy among all the damage types of CFRP, this research demonstrates the ability of the PSFBG-based ultrasonic sensor in AE detection. Analyses in both the time and frequency domains were performed to confirm that the AE signal detected by the PSFBG was analogous to that detected by the PZT sensor. Furthermore, position identification of multiple transverse cracks was achieved according to different ToFs [132].

Because the AE signal detected by PSFBG can be represented in high quality, a more precise analysis based on the mode property of the Lamb wave rather than traditional statistical analysis can be conducted. Damage types can be discriminated based on the relations between the wave modes and the corresponding strain [129], or the modes and the peak frequency [133]. It was also found that two quantitative parameters, namely the amplitude ratios of the S_0_ and A_0_ modes from AE signals and the peak frequency, can be used to discriminate AE signals that correspond to transverse cracks, delaminations, and fiber breakages. Furthermore, the behaviors of the S_0_ and A_0_ modes of the AE signals that are detected by PSFBG have been related to the difference between the AE source orientation of the transverse crack and that of delamination [134].

## 6. Future Trends

Currently, mainstream ultrasonic SHM still uses piezo sensors because of the vast experience accumulated in the decades of experiments and applications after the piezo sensor was first designed in the late 1940s. End-users in various industrial sectors are confident and comfortable to use this widely accepted sensor that has plenty of accessories and instruments, although the sensors themselves have several crucial problems. Therefore, although FBG-based sensing systems have been developed and can precisely detect ultrasonic wave propagating in material structures, time and effort are needed to introduce this technique and let end-users accept it. More importantly, the key problems of FBG ultrasonic sensors that hinder their usage in a wider range of applications, including truly multiplying for simultaneous ultrasonic detection in multiple channels, resistance to environmental disturbances, further improvements in sensitivity and bandwidth, and the high cost of the demodulation system, should be properly solved by using new techniques.

The performance of an FBG sensor can be further improved by using different fiber materials and by designing different sensor heads. Polymers, such as poly (methyl methacrylate) (PMMA), are potential fiber materials for ultrasonic detection. For example, a six-times ultrasonic sensitivity improvement is achieved using an FBG written in a PMMA fiber, which arises as a result of the much more compliant nature of PMMA compared to silica in water [22]. Multifunctional sensor heads developed by integrating an FBG with other in-fiber devices, like a Fabry-Perot interferometer [135,136] or a Mach–Zehnder interferometer [137], can achieve temperature, strain, and ultrasonic detection to facilitate more comprehensive SHM analyses. For example, the temperature information is helpful for modifying the baseline shift in ultrasonic SHM [138].

Using demodulation techniques based on lasers (Section 3.3, Section 3.4 and Section 3.5) improves the sensitivity but usually sacrifices multiplexing. This is because one laser only can power one sensor if a controller is used to tune the wavelength of the laser. However, multiplexing is a fundamental requirement for position identification in passive ultrasonic detection, namely, impact detection and AE detection, as the ultrasonic signal is not repeatable. One solution to multiplexing is using multiple laser light sources that have a low cost [139]. The other solution is seeking an advanced demodulation technique. For example, the system using a pulsed laser claims to have a multiplexing ability due to its synergy of the advantages of laser source and broadband light source [140]. Another problem in the conventional sensing system is the robustness, i.e., its resistance to environmental disturbance like temperature. Especially when a PSFBG with narrow FWHM is used, precisely tuning the laser wavelength is challenging. In fact, multiplexing and robustness are conflicting requirements because the feedback controller negatively affects multiplexing and multiplexing usually sacrifices robustness.

When FBG is used in acousto-ultrasonic sensing, PZT or MFC plays as a transducer to convert the electric signal to a mechanical vibration that currently cannot be achieved using optical fibers because optical fibers do not have the capability of electromechanical conversion. To a certain extent, the claimed advantages of optical fiber sensors, such as the embedding ability and immunity of electromagnetic interference, become weaker in acousto-ultrasonic detection since piezoelectric ceramics are still necessary. One possible way to achieve all-optical acousto-ultrasonic detection is using techniques based on laser ultrasonics [141]. Ultrasound is generated by the sudden thermal expansion in a thermoelastic regime when a tiny surface of the material is heated by a high-power and short-pulse laser. Traditionally, laser ultrasonics is a non-contact technique for nondestructive testing and it needs bulky instruments. But now, it is possible to minimize the size of the instruments if a metal on the tip of a fiber absorbs the pulsed light energy and converts it into an ultrasonic signal. Several prototypes of optical fiber actuators have been proposed and demonstrated [142,143,144]. These breakthroughs of optical fiber ultrasonic actuators give us directions for using the same principle to generate Lamb ultrasonic waves in a plate-like structure.

Besides three typical applications introduced in Section 5, FBG also can be potentially used in more advanced applications. A demanding application is nonlinear ultrasonic sensing for micro-damage evaluation, such as metal fatigue and fatigue-induced cracks that are barely visible [145]. It can also be utilized in harsh environments thanks to its unique characteristics. For example, it can replace conventional sensors in nuclear power plants because of its high resistance to radiation, chemical contamination, and electromagnetic interference [146]. Furthermore, a remote sensing structure can be used to guide the ultrasonic wave out from an environment that is over 1100 Celsius and subsequently detect it by using PSFBG [100]. By further incorporating FBG with other techniques such as sapphire fiber [147], FBG can possibly detect ultrasonic signals in temperatures over 1800 Celsius, which will have a significant impact on the aerospace industry.

## 7. Conclusions

FBG-based ultrasonic sensing systems and their applications in ultrasonic SHM have been reviewed in this paper. To provide precise information for structural analysis, it is necessary not only to carefully and comprehensively consider the sensor head, demodulation system, and installation method but also to address the practical problems of directional sensitivity, environmental disturbance, multiplexing requirement, and data processing. After decades of development, the performance of the FBG ultrasonic sensor has been considerably improved. In addition to impact detection and acousto-ultrasonic detection that were achieved earlier, precise AE detection has been achieved recently. Various targets of SHM, including position identification, damage evaluation, and defect discrimination, have been accomplished by FBG ultrasonic sensors, indicating its comparable ability to the conventional PZT sensor.

However, whether this pace of development can be sustained and can eventually lead to the widespread use of FBG in ultrasonic SHM depends on whether several key problems can be overcome, such as the multiplexing requirement, the resistance to environmental disturbances, and the high cost of the system. Although several new techniques have been demonstrated in the laboratory, additional effort is needed to put these designs into practice. In addition, the end-users should select the most suitable sensing system according to their core needs because FBG-based ultrasonic SHM is not a universal technique and is not suitable for use under all conditions.

## Figures and Tables

**Table 1:** The commercial FBG sensors and their specifications.

Manufacturer	Model	Frequency Property (kHz)	Resolution Property	Wavelength Accuracy	Application
Ibsen Photonics A/S	I-MON 256 HS	35 (Measurement frequency)	<0.5 pm	5 pm	impact
Smartfibres Inc.	SmartScan	25 (Scan Frequency)		<5 pm	impact
Redondo Optics, Inc.	FBG-Transceiver^TM^-500	20 (Sampling rate)	5 pm	5 pm	impact
	FAESense^TM^	300 (AE frequency)	0.1 με/Hz		ultrasonic
Intelligent Fiber Optic Systems Corporation	I*Sense^®^ HS48M	maximal 5000 (detection speed)	0.1 pm	2 pm	ultrasonic

**Table 2:** The demodulation techniques and their characteristics.

Method	Light Source	Stage	Main Characteristics
1	Broadband light source	In practice	Fair sensitivity, multiplexing, low cost
2	Laser light source	In practice	High sensitivity, weak multiplexing, need feedback controller
3	EDFL	In laboratory	Temperature and strain resistance, frequency-dependent sensitivity, weak multiplexing
4	Modulated laser	In laboratory	High sensitivity, temperature and strain resistance, high cost, no multiplexing possibility

**Table 3:** The summarization of FBG sensor, demodulation, and installation in three different ultrasonic SHM applications.

Application	Sensor		Demodulation				Installation			Reference
	FBG	PSFBG	Broadband Light	Laser Light	EDFL	Modulated Laser	Surface Mounted	Ultrasonic Waveguide	Mechanical Assist	
**Impact**	X		X				X			44, 45, 84, 107, 112
	X			X			X			105, 111, 139
	X			X					X	28
**Acousto-** **ultrasonic**	X		X				X			27, 49, 50, 87, 115, 117–120, 122
	X			X			X			25, 29, 36, 42, 80, 86, 97, 114, 117, 121
	X			X				X		36, 92, 93, 97, 98
	X			X					X	28, 36, 88, 101
	X				X		X			63-66
		X	X				X			40, 52
		X		X			X			57, 58, 60
		X			X			X		67, 69
		X				X	X			73, 74
**Acoustic** **emission**	X			X			X			46, 125
		X		X			X			127
		X		X				X		96, 99, 128–133
